# [Retracted] microRNA‑7 regulates cell growth, migration and invasion via direct targeting of PAK1 in thyroid cancer

**DOI:** 10.3892/mmr.2023.12992

**Published:** 2023-04-03

**Authors:** Kai Yue, Xudong Wang, Yansheng Wu, Xuan Zhou, Qinghua He, Yuansheng Duan

Mol Med Rep 14: 2127–2134, 2016; DOI: 10.3892/mmr.2016.5477

Following the publication of this paper, it was drawn to the Editor's attention by a concerned reader that a data panel portraying cell migration and invasion assay data shown in Fig. 7C was strikingly similar to a panel that had appeared in another article by different authors at a different research institute, which had been submitted for publication earlier than the submission date of this article. Moreover, a large number of overlapping data panels were identified comparing the data in Figs. 4A and B and 7C and D. Owing to the fact that the contentious data in Fig. 7C in the above article were already under consideration for publication prior to its submission to *Molecular Medicine Reports*, the Editor has decided that this paper should be retracted from the Journal. The authors were asked for an explanation to account for these concerns, but the Editorial Office did not receive a reply. The Editor apologizes to the readership for any inconvenience caused.

